# Unpredictable spillovers among water uses? An analysis of agricultural, industrial, and household uses of water in the Balkans

**DOI:** 10.1371/journal.pone.0235079

**Published:** 2020-07-02

**Authors:** Chiara Natalie Focacci, Alberto Quintavalla

**Affiliations:** 1 Department of Economics, University of Bologna, Bologna, Italy; 2 Rotterdam Institute of Law & Economics, Erasmus University Rotterdam, Rotterdam, The Netherlands; Institute for Advanced Sustainability Studies, GERMANY

## Abstract

In times of water shortage, it becomes increasingly relevant for policymakers to understand the existing relationships between different types of water use, so as to encourage efficient water management. This article makes use of yearly data on agricultural, industrial, and household water use in the Balkan countries of Bulgaria, Romania, and Serbia. It does so to identify the potential interactions among these three categories of water use. Using a deterministic model based on differential equations, we provide an analysis of the interactions among these different sectors of water use for the period between 2008 and 2017. Results show that interactions among these different categories do not remain constant over periods of time, either across or within the countries analysed. We find that, for most countries, industrial and household water uses are more likely to be characterised by mutualism and competition, instead of a predator-prey relationship. Agricultural water use, on the other hand, takes on the role of predator against the other two.

## Introduction

Because water is a scarce resource, neither groundwater nor surface water is always sufficient to meet water demands. This is even more true for the era we live in, where water scarcity has exacerbated progressively due to a number of factors, such as growing populations, accelerating climate change, and increased standards of living [1]. In this vein, it is expected that by 2025 more than 60% of the global population will suffer from water scarcity in some shape or form [2]. This is set to be an issue many governments will face. For this reason, an understanding of how water resources can be allocated among different sectors will be increasingly relevant.

On this subject, a large body of scientific literature has investigated how to allocate water resources efficiently [3]. To address this challenge, existing studies mostly focus on the future projections of water demands as well as on the determinants of these said demands [4–6]. A common feature of these studies is the underlying existence of interactions among the different use. In other words, it is contended that an increase of water use in a defined sector is likely to affect, either positively or negatively, another water sector. For instance, a hypothesis advanced by some of the literature is that industrial and household water use are in a relationship of competition to agricultural water use [7–9]. An increase in urbanisation, and the consequent expansion of industries, alongside the rising standards of living would account for a reduction in agricultural water use to the advantage of water consumption in the household and industrial sectors [10–13].

Nonetheless, it can be noted that there is a lack of studies which address the subject from a quantitative perspective. Using a deterministic model based on differential equations, this article aims to address this overlooked issue. Specifically, it examines the type and intensity of relationships that exist in water uses among the industrial, agricultural, and household sectors in three different countries, namely Bulgaria, Romania, and Serbia. Findings show that the relationships among their different sectors vary considerably over periods of time between predator-prey, mutualism and competition.

These findings can be important for policymakers, especially in the context of the challenges of coming years. For example, as was highlighted by [14] recently, the impact of climate change on water distribution will be significant in the next few decades, while the demands for urban water will rise by 80%. National policies will have to address these unavoidable issues, by putting into place efficient management schemes. To do that, policymakers will need a good understanding of how the different sectors of water interact. By providing an analysis of how changes in water use of one sector can affect the other two (and vice versa), this paper aims to facilitate this task.

## Materials & methods

To perform the analysis, we collect aggregate data on yearly water use in Bulgaria, Romania, and Serbia, for the period 2008-2017. All the data is transformed into shares, obtained by dividing the amount of water used in each category by the sum of all water used in the three categories. The data is analysed by applying a deterministic model based on differential equations developed in [15]. For an accurate description of the model and the relative formal proof, we refer the reader to [15], [16], and [17]. The model was originally designed to analyse the interactions among competitive firms [18], but it was applied later to an array of contexts such as the study of interactions among renewable energy sources and oil prices [19], inter-port interactions [20] and tourism dynamics [16]. Indeed, the model aims to examine how entities interact to allocate a scarce resource in an established niche. It is therefore applicable to our present purposes.

In this article, we use it to investigate how the agricultural, household and industrial sectors interact in terms of water use. The fact that an increase of water use in a certain sector positively or negatively influences the water use in another sector has been a common underlying feature of many studies on water allocation [7, 9, 12]. Thus, this model is particularly apt to capture any type of interaction that occurs in a context where a change in a factor related to one category (e.g. agricultural water use) affects other categories (e.g. household water use) with which it is interacting.

In particular, we refer to the sign of the potential spillovers between water uses as interaction coefficients. These represent how an increase (or decrease) in the power of an entity, such as agricultural water use, influences the power of another entity, such as industrial water use. The sign of the interaction coefficients represents the sign of type of spillovers that exist between different entities, or water uses (see Table 1).

**Table 1:** Types of interactions among water uses.

*g*_*i*_	*g*_*j*_	Type of Interaction	Description
+	+	Competition	An increase (reduction) in a certain water use negatively (positively) affects another water use.
−	+	Predator-Prey	An increase (decrease) in water use A negatively (positively) affects water use B (prey). An increase (decrease) in water use B positively (negatively) affects water use A (predator).
−	−	Mutualism	An increase (a reduction) in a certain water use increases (reduces) another water use.
−	0	Commensalism	An increase (decrease) in water use A positively (negatively) affects water use B. Water use A is unaffected by changes in water use B.
+	0	Amensalism	An increase (decrease) in water use A negatively (positively) affects water use B. Water use A is unaffected by changes in water use B.
0	0	Neutralism	There is no interaction.

The table explains the possible relationships that exist between the different water uses. Particularly, it illustrates the concepts of competition, mutualism, predator-prey, amensalism, commensalism, and neutralism by looking at the sign of the interactions among different entities. The source for this table is [18].

Besides this first important property, the use of a deterministic model based on differential equations presents three other substantial advantages for the task at hand. First, because interaction coefficients are dependent on time, our model allows us to capture significant structural changes. This turns out to be crucial, given the prominent role of contextual factors in water use interactions. In other words, a large number of studies show that exogenous factors (e.g. technology, urbanisation and rising income) can strongly affect the allocation of water among different sectors [1], and thus their changes over time. Second, the model analyses the *simultaneous* interactions among three water uses, without the need to isolate them into pairs. In other words, it captures the specific kind of interactions, taking into account all three sectors considered. This property becomes advantageous in cases where multiple sectors coexist in the allocation of a scarce resource. Third, because the solutions of the model are known we do not have to estimate the parameters using expensive numerical methods; in other words, this empirical strategy is not data demanding. Instead, the roles played by the different sectors can be derived directly from the data at hand.

In particular, as in [18] we define each share of water use in the form of a logit model explained below, so that the shares of the *i*th use are, respectively,

$$\begin{array}{c} \begin{array}{c} x_{i}\left(t\right)=\frac{\text{exp}(f_{i}(t))}{1+\sum_{j=1}^{3}\text{exp}\left(f_{j}(t)\right)},\;i=1,2,3\\ x_{0}\left(t\right)=\frac{1}{1+\sum_{j=1}^{3}\text{exp}\left(f_{j}(t)\right)}, \end{array}\;\forall t\geq t_{0}, \end{array}$$ (1)

where the utility function of a certain type of water use *f*_*i*_(*t*), *i* = 1, …, 3, such as agricultural, industrial, or household water use is equal to:

$$\begin{array}{c} f_{i}(t)=\ln(\frac{TS_{i}(t)}{TS_{0}(t)}),\;i=1,2,3. \end{array}$$ (2)

If all the utility functions *f*_*i*_(*t*) are of class *C*^2^([*t*_0_, + ∞)), then Eq (1)_1_ are the unique (global) solution of the Cauchy problem as in:

$$\begin{array}{c} \left\{ \begin{array}{c} {\dot{x}}_{i}\left(t\right)=g_{i}(t)x_{i}\left(t\right)[1-x_{i}\left(t\right)]-\sum_{j=1,j\neq i}^{3}g_{j}(t)x_{j}\left(t\right)x_{i}\left(t\right),\;i=1,2,3\\ x_{i}\left(t_{0}\right)=\frac{\text{exp}(f_{i}(t_{0}))}{1+\sum_{j=1}^{N}\text{exp}\left(f_{j}(t_{0})\right)} \end{array}t\in [t_{0},+\infty ),\right. \end{array}$$ (3)

where ${\dot{x}}_{i}\left(t\right)=dx_{i}\left(t\right)/dt$, $x_{0}\left(t\right)=1-\sum_{i=1}^{3}x_{i}\left(t\right)$, and

$$\begin{array}{c} g_{i}(t)={\dot{f}}_{i}\left(t\right)=\frac{{\dot{TS}}_{i}\left(t\right)}{TS_{i}\left(t\right)}-\frac{{\dot{TS}}_{0}\left(t\right)}{TS_{0}\left(t\right)},\;i=1,2,3. \end{array}$$ (4)

Because the deterministic model based on differential equations describes the interaction between the *i*th and *j*th water uses, the water share of interest depends on the logistic growth rate function *g*_*i*_(*t*) and the interaction functions *g*_*j*_(*t*) between the *i*th and *j*th use of water. The maximum capacity of each water use is equal to one. The type of interaction among the different uses of water is determined by the sign of the functions *g*_*i*_(*t*) and *g*_*j*_(*t*), while the utility functions *f*_*i*_(*t*) are defined as the non-linear combination of time-varying variables *V*_*h*_, *h* = 1, …, *M*,. In particular, the share of the *i*th use of water increases when its utility function *f*_*i*_(*t*) increases, while it decreases when the utility function *f*_*j*_(*t*) of any other use of water increases. Eq (3) allows us to evaluate how changes in the utility functions affect the respective shares of water use. Thus, the actual available data will be based on the shares rather than on the utility functions, which are determined from the data on shares available over time via a fitting procedure [18]. As is standard for the mathematical literature, the latter is performed using a Fourier series [18].

Using the deterministic model of differential equations, we are able to capture all the potential types of interactions among water use. Although water is a scarce resource, this does not imply that only competitive interactions among different entities may exist. First of all, and as shown by previous literature, there are certain instances where two water sectors can have a positive relationship, even in times of water scarcity. For example, urbanisation coupled with higher living standards tends to cause mutualism in industrial and urban water uses. Indeed, the initial stage of industrialisation characterised by a rather large amount of consumed water tends to bring higher standards of living and, accordingly, increase water consumption for household purposes [8, 11, 21]. Secondly, water demands are not always higher than water availability at a given time. In this case, all water demands can coexist without resulting in competitive interactions [6]. The possible types of relationships are reported in Table 1, which illustrates the mechanisms of competition, predator-prey, mutualism, commensalism, amensalism, and neutralism.

With respect to the accuracy of the model, we assess the accuracy and reliability of our model exploiting the standard measure of error MAPE, or the Mean Absolute Percentage Error. In particular, following [22], we consider our model to be highly accurate when *MAPE* < 10%; good if 10% < *MAPE* < 20%; reasonable when 10% < *MAPE* < 50%; and inaccurate if *MAPE* > 50%.

Our Mean Absolute Percentage Errors are calculated as in:

$$\begin{array}{c} MAPE=\frac{1}{n}\sum_{i=1}^{n}|\frac{h_{i}-p_{i}}{h_{i}}|100\%, \end{array}$$ (5)

where *h*_*i*_ and *p*_*i*_ are respectively the historical and predicted values.

The model also features an outside option, or an entity with an inactive role in the niche considered in this article. In particular, this is an entity that does not directly compete with the categories of water use considered [23]. Thus, the outside option is selected from subjects who are not interested in either industrial, agricultural, or household water use. In this example, an outside option in the context of water use could be environmental water use, since it does not have a directly active role and is only used selectively by subjects not associated with industrial, agricultural, or household water use. In other words, this fourth category of water use can be contextualised as a residual share in the sphere of water uses—by residual we imply that this category of water use is external to the interactions between agricultural, industrial, and household sectors. This outside option, whose utility function is equal to zero, is necessary so as not to alter the mathematical results.

Data is collected from Eurostat at the aggregate yearly level from 2008 to 2017 and refer to the different uses of water. The consumption of water varies considerably among different sectors. According to an AQUASTAT report analysed by [2], 95 percent of water supply is usually used for irrigation purposes. Yet, the specific amounts may vary over region or within the same country. This is, for instance, the case in China, where the more rural Western provinces consumes larger amounts of water for agricultural purposes than their Eastern compatriots [24]. Having said this, in this article we refer to the three main water consumption sectors recognised by researchers. In particular, we distinguish between three different categories of water use; namely, the agricultural use of water, the industrial use of water, and the household use of water. Indeed, since this article aims to identify the types of interactions among water uses in a bid to help allocate water resources more efficiently, focusing on the three water sectors that consume the most water seems a reasonable choice. Therefore, in this analysis we do not account for secondary water uses such as the ecological uses of water [24] or the recreational uses of water [13].

To study the interactions between the different categories cited, we use water abstraction for household purposes; water abstraction for agriculture; and water abstraction for the manufacturing industry. Our categories of water use are measured by million cubic meters and refer to annual freshwater abstraction for, respectively, agriculture, industry, and households. For a better understanding of water definitions, it is important to stress that our data refers to water abstraction from both groundwater and surface water, and does not directly include water consumption.

Indeed, water use, water withdrawal, and water consumption are three definition with a different meaning. Water use refers to the amount of water that a given sector, such as the industrial sector, actually *consumes*, and so therefore encompasses both water withdrawal, water consumption, and return flows. Water withdrawal is the most commonly used indicator of reference for water research, and indicates the quantity of water abstracted from groundwater and surface water. Water consumption refers to the volume of water abstracted that is ‘evaporated, transpired, incorporated into products or crops, or consumed by humans’ [10]. Studying the interactions that exist among water uses can therefore be helpful only when we account for the actual abstraction of water destined to different uses regardless of the actual consumption and return flows. In this article, we make use of data that refers to the abstraction of water for a series of reasons. First, data is easier to retrieve. Second, providing an analysis on water abstraction is significantly useful for policymakers to make more informed decisions in terms of water allocation. Our results can be used by policymakers to understand how much of the water should be abstracted, how much should be allocated; and, finally, in what ways.

Due to availability and continuity of data over time, we give priority to the countries in question; namely, Bulgaria, Romania, and Serbia. Most importantly, in our analysis we give priority to these countries due to the fact that they are part of the same geographical area, and they are all crossed by the River Danube, which represents a large, albeit slightly polluted, source of freshwater resources. Given that water management is highly context-dependent, it seems reasonable to focus on one geographical area, characterised by states that have all similar access to water resources, and therefore can be compared, making it easier to identify common trends. Furthermore, Bulgaria, Romania, and Serbia have concluded various bilateral and multilateral agreements for water uses among the three states [25], including the Convention on the Cooperation for the Danube Protection and Sustainable Use. Together with Northern Macedonia, the aforementioned three countries are also the countries that have the lowest level of availability of water per person in Southeast Europe [26]. The fact that these countries have an even more limited availability makes our analysis even more interesting and pertinent. This is also explained by the literature according to which scarcity necessarily implies a greater degree of competition among various sectors [27].

## Results & discussion

**Fig 1:**
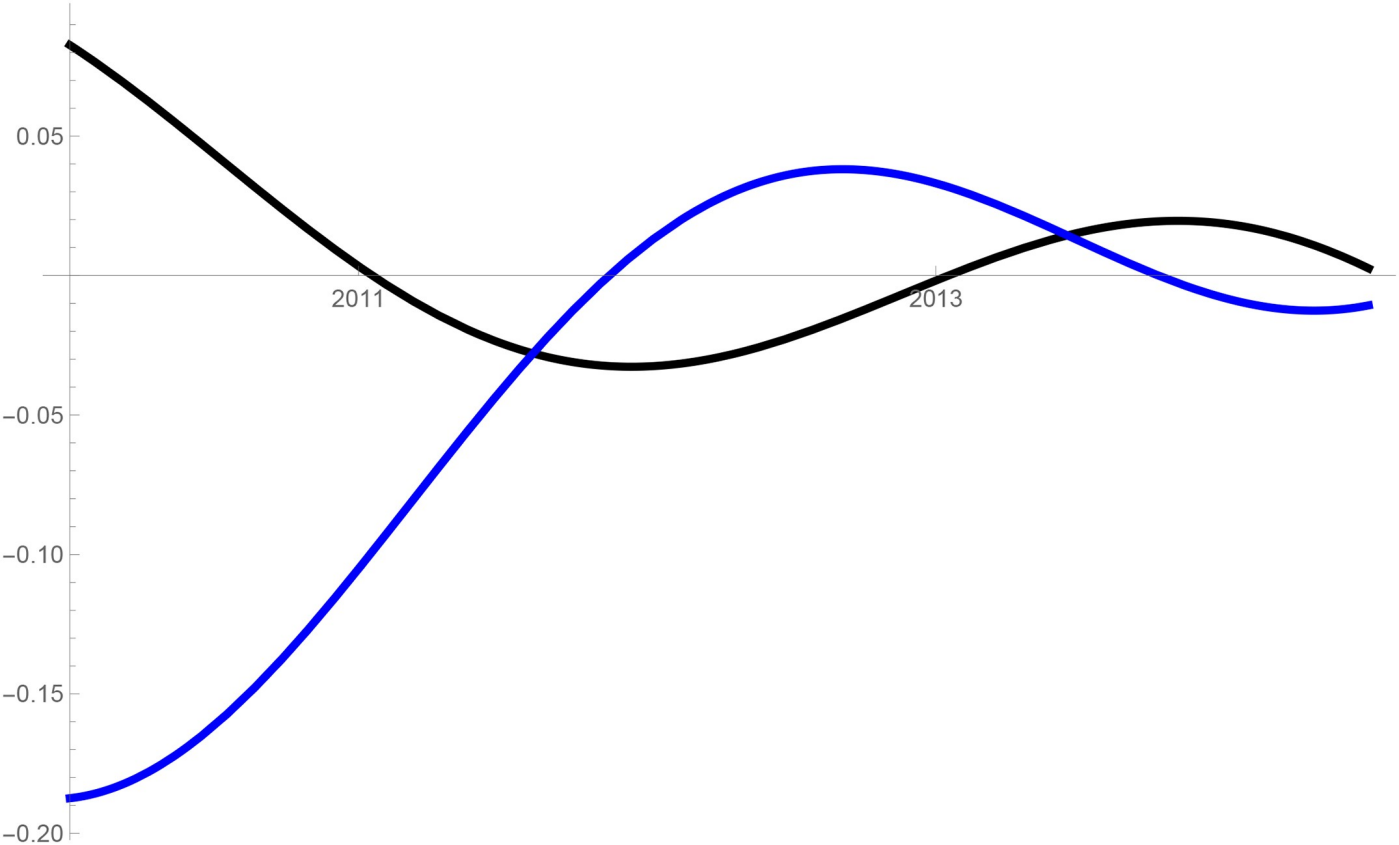
Industrial and agricultural water use in Bulgaria (2008-17).

**Fig 2:**
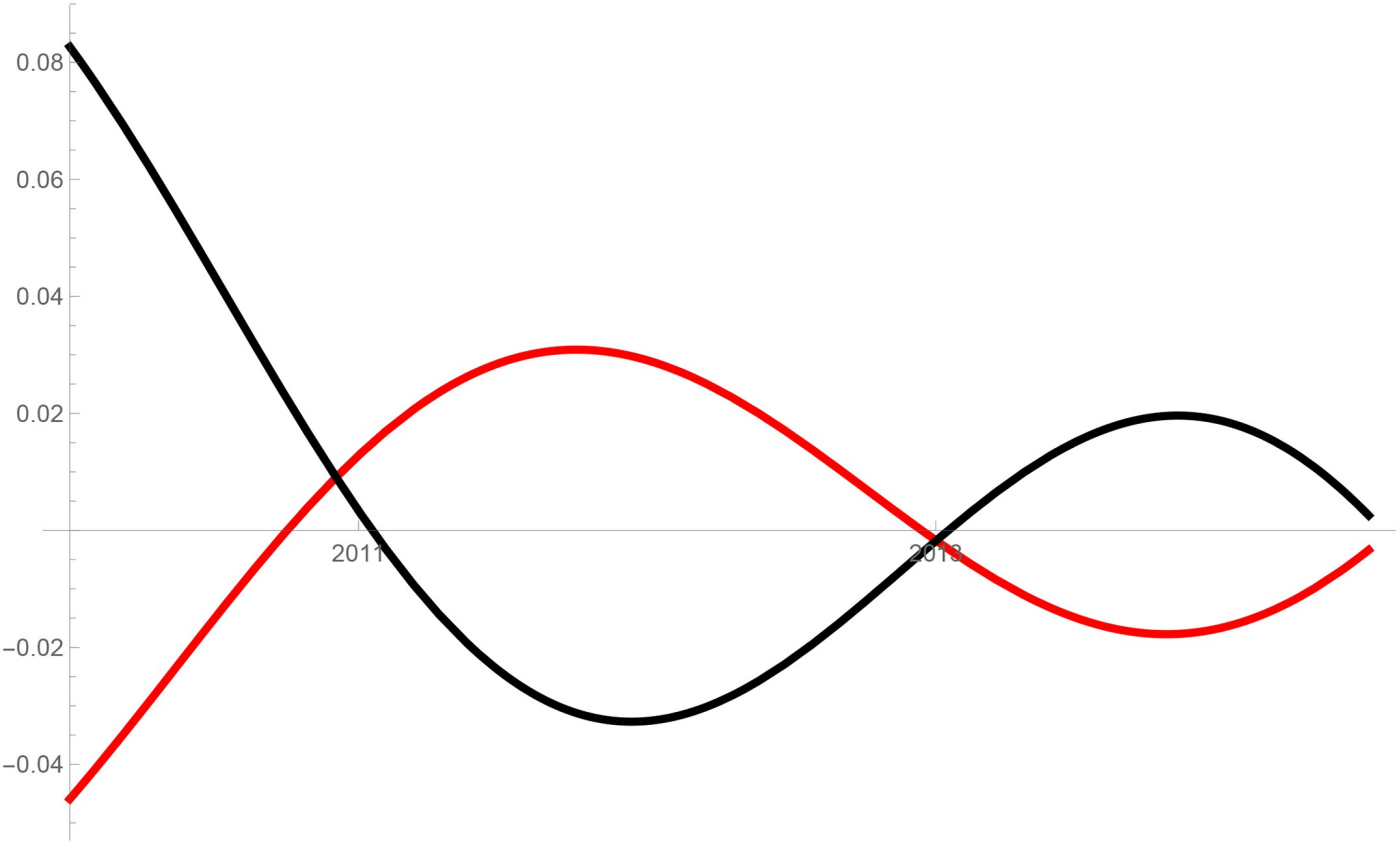
Agricultural and household water use in Bulgaria (2008-17).

**Fig 3:**
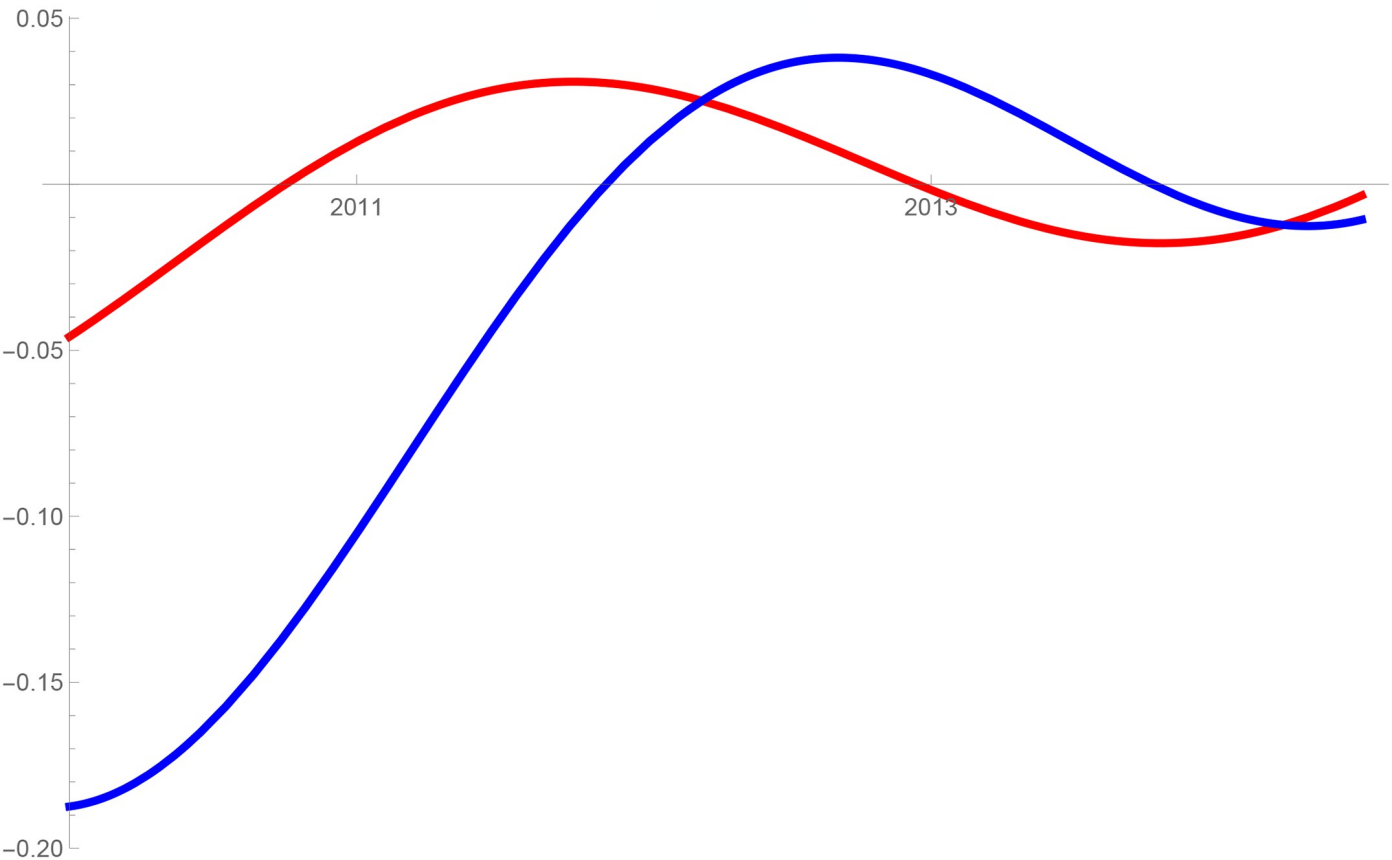
Industrial and household water use in Bulgaria (2008-17).

**Fig 4:**
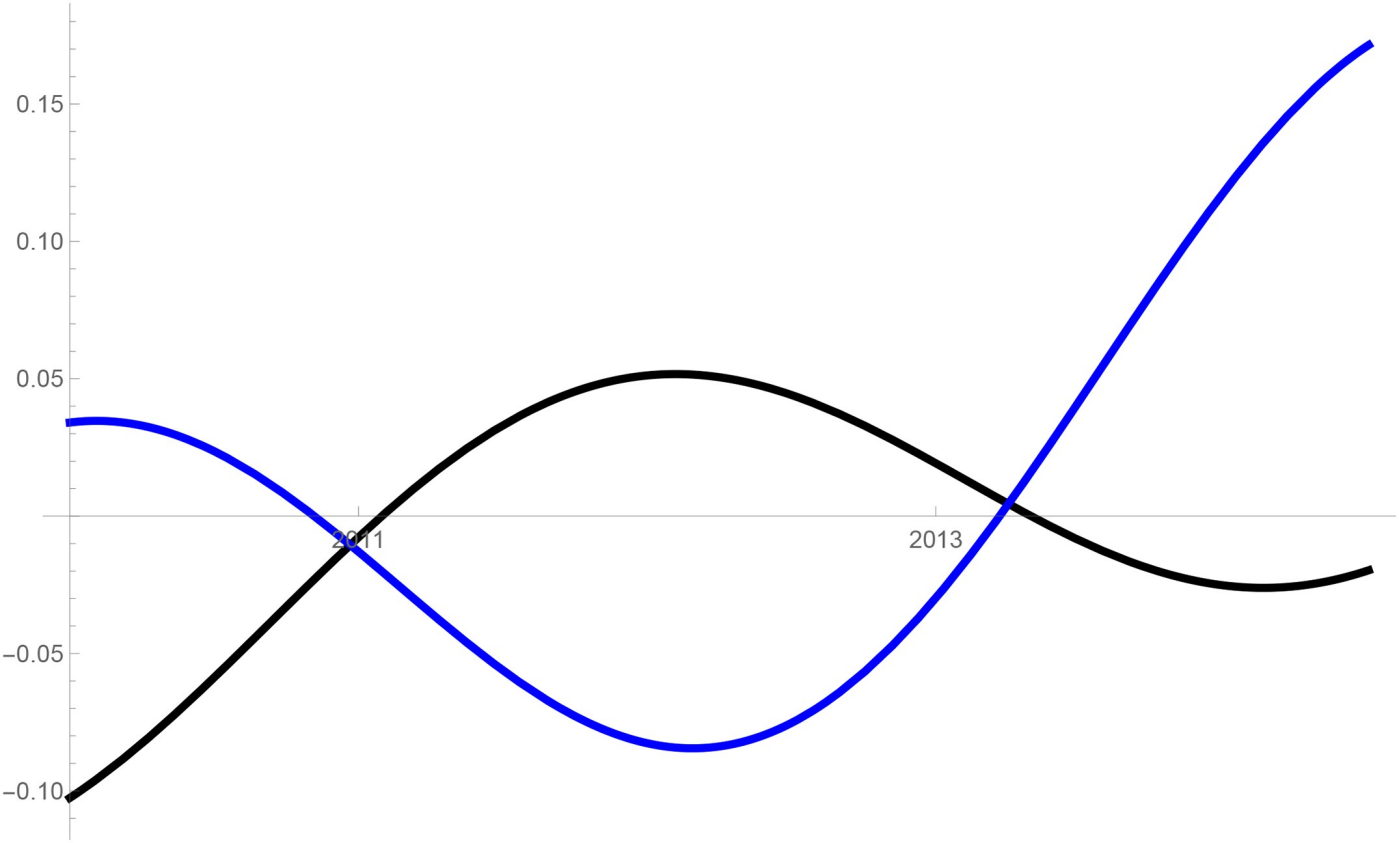
Industrial and agricultural water use in Serbia (2008-17).

**Fig 5:**
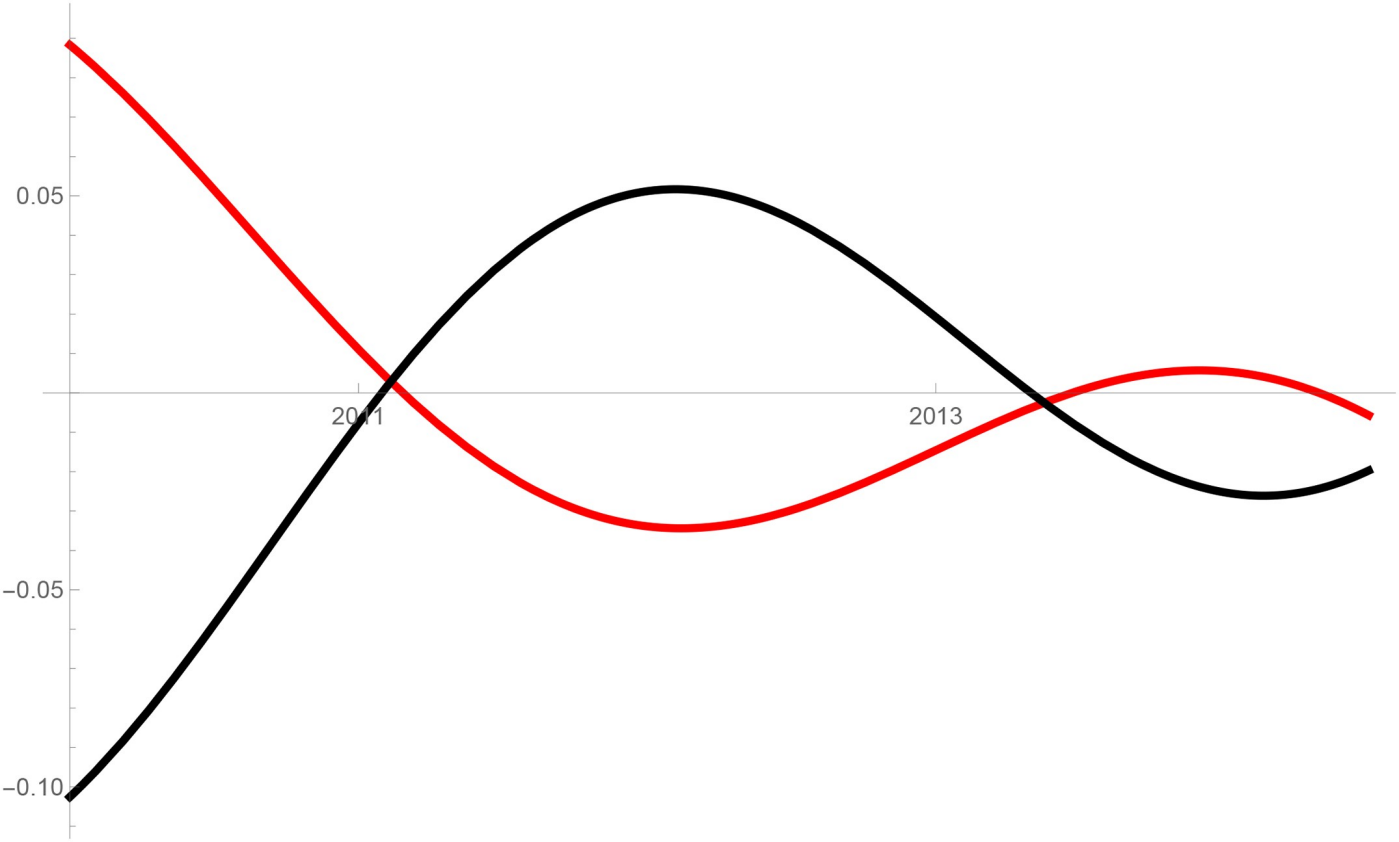
Agricultural and household water use in Serbia (2008-17).

**Fig 6:**
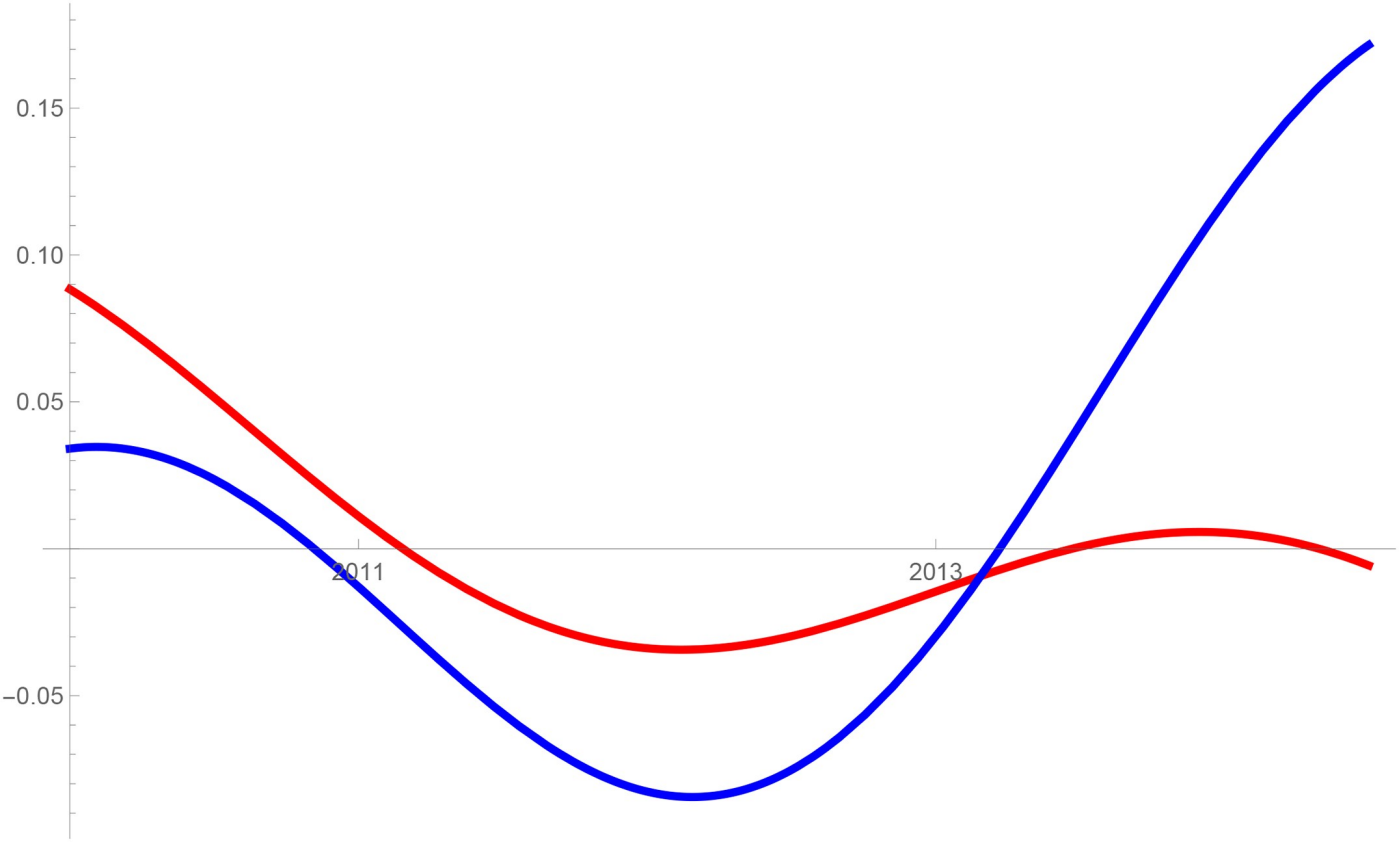
Industrial and household water use in Serbia (2008-17).

**Fig 7:**
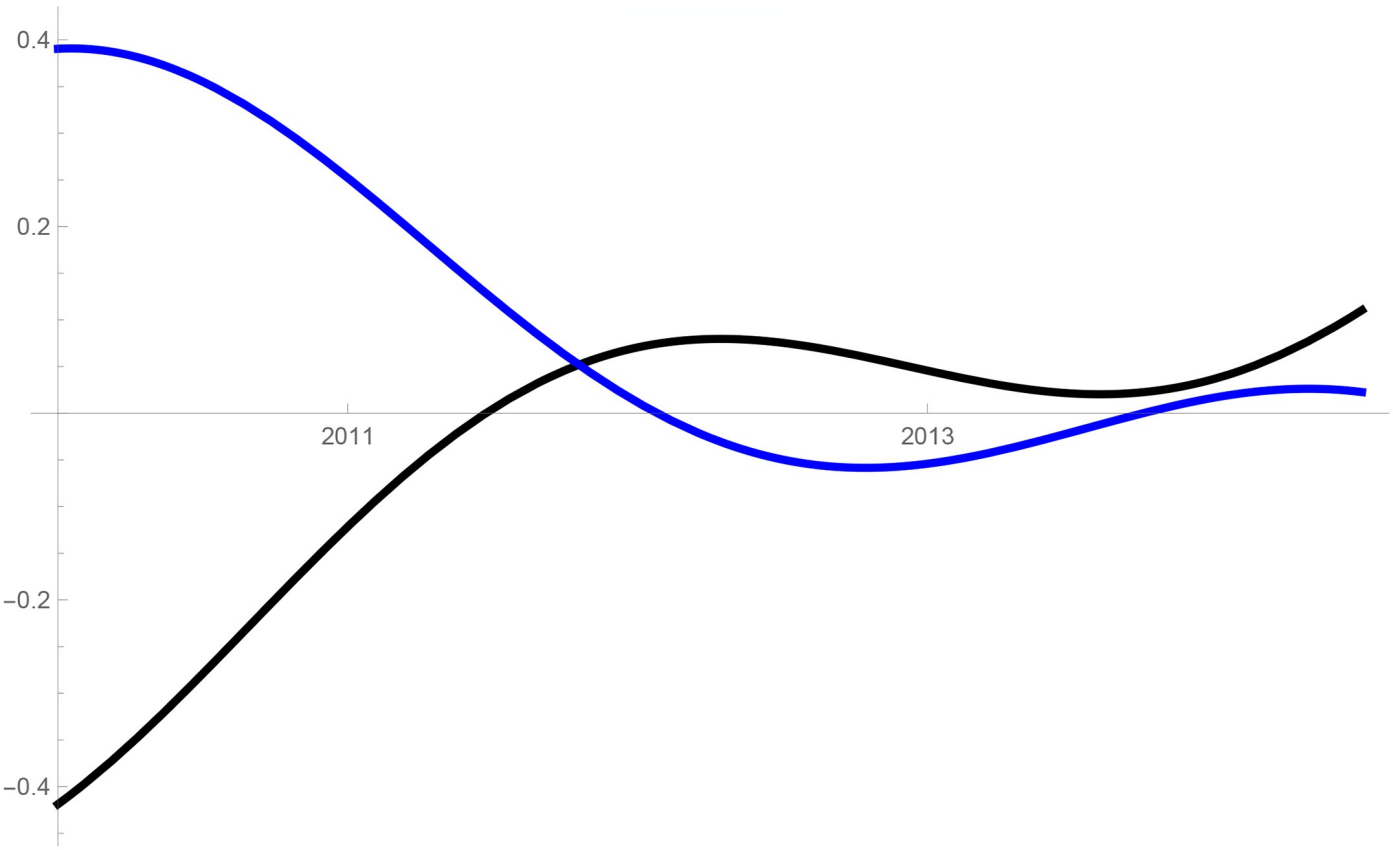
Industrial and agricultural water use in Romania (2008-17).

**Fig 8:**
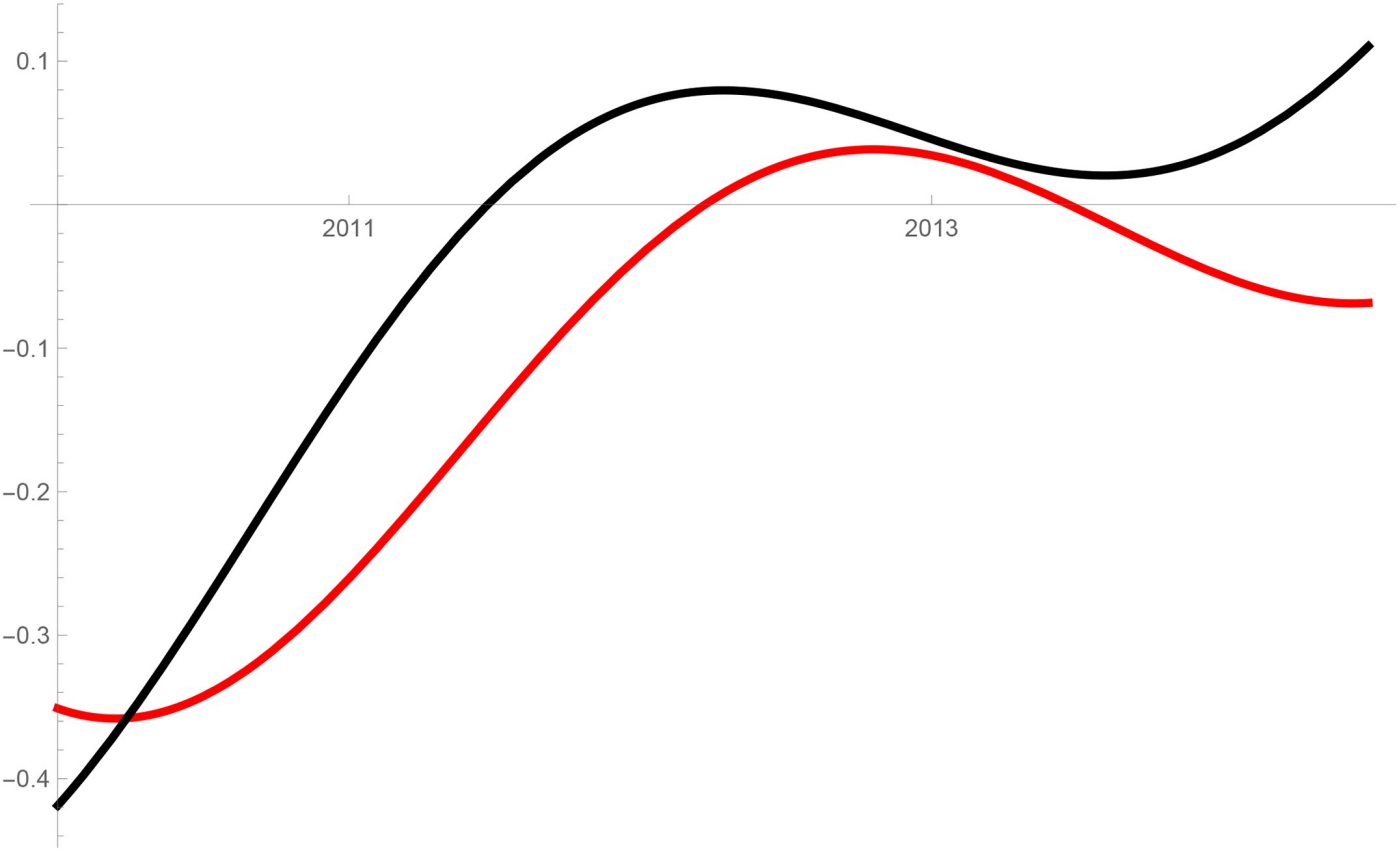
Agricultural and household water use in Romania (2008-17).

**Fig 9:**
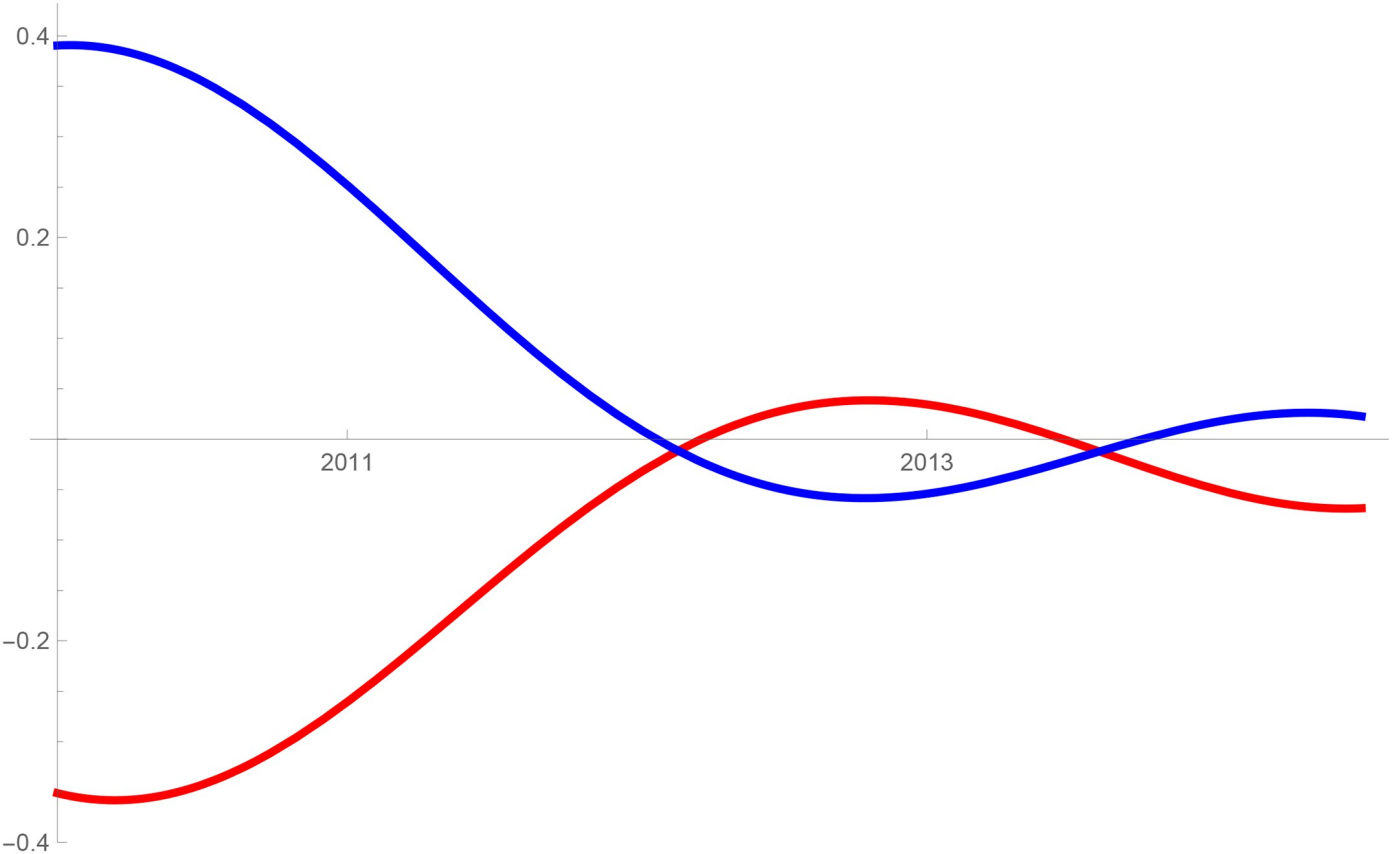
Industrial and household water use in Romania (2008-17).

Figs 1–9 provide a graphical illustration of the interaction coefficients between the different shares of agricultural, industrial, and household water uses in the Balkan countries of Bulgaria, Romania, and Serbia over time. Years are shown in the horizontal axis for the period 2008-17, while interaction coefficients are shown in the vertical axis. In particular, the red curve represents the interaction coefficients related to household water use; the blue curve represents the interaction coefficients related to industrial water use; and the black curve represents the interaction coefficients related to agricultural water use. Because the type and intensity of interactions among different water uses changes over time, any claim that there is one dominant type of relationship in absolute terms is erroneous.

The figures are built based on the shares of water used described in Tables 2, 3 and 4. While the tables illustrate the historical and observed shares of water use in the three countries, and the three categories, Figs 1–9 the interaction coefficients. In other words, the figures represent the interaction coefficients of the sectors of water use analysed. Descriptive statistics are provided in Tables 5, 6 and 7. In line with the existing literature, it is possible to note that shares of water use have not remained stable in any country under investigation over periods of time. This is, for instance, readily noticeable in Romania where the shares of water use fluctuated significantly between 2008 and 2017. For example, there is a steep increase in the share of water use for industrial purposes during the first years under investigation. On the other hand, unlike in Romania, the shares of water uses for household and agricultural purposes are relatively high in both Bulgaria and Serbia. This observation suggests that while Romania is characterised by water-intensive industries, Bulgarians and Serbians tend to abstract fresh water for irrigation and domestic activities.

**Table 2:** Shares of household, agricultural, and industrial water use in Bulgaria (2008-17).

Household Water Use	Agricultural Water Use	Industrial Water Use	*Year of reference*
45.3	45.0	9.7	2008
45.7	46.6	7.7	2009
45.9	46.5	7.6	2010
43.6	49.9	6.5	2011
46.5	47.3	6.2	2012
49.1	44.4	6.5	2013
50.2	42.6	7.2	2014
47.4	45.2	7.4	2015
45.9	47.6	6.5	2016
47.7	45.1	7.2	2017

The table shows the shares of water use for household, agricultural, and industrial purposes in Bulgaria between 2008 and 2017. Data is obtained by dividing the amount of water used in each category by the sum of all water used in the three categories and is measured by million cubic meters and refer to annual freshwater abstraction for household, agricultural, and industrial water use. The vertical axis refers to the interaction coefficients.

**Table 3:** Shares of household, agricultural, and industrial water use in Romania (2008-17).

Agricultural Water Use	Industrial Water Use	Household Water Use	*Year of reference*
41.9	29.2	28.9	2008
41.2	32.1	26.7	2009
19.3	13.9	66.8	2010
18.6	17.9	63.5	2011
19.5	20.4	60.1	2012
17.9	20.9	61.2	2013
18.4	20.1	61.5	2014
17.9	22.6	59.5	2015
18.4	21.9	59.7	2016
17.2	24.9	57.9	2017

The table shows the shares of water used for household, agricultural, and industrial purposes in Romania between 2008 and 2017. Data is obtained by dividing the amount of water used in each category by the sum of all water used in the three categories and is measured by million cubic meters and refer to annual freshwater abstraction for household, agricultural, and industrial water use. The vertical axis refers to the interaction coefficients.

**Table 4:** Shares of household, agricultural, and industrial water use in Serbia (2008-17).

Household Water Use	AgriculturalWater Use	Industrial Water Use	*Year of reference*
44.3	46.9	8.8	2008
50.5	40.7	8.8	2009
48.4	43.1	8.5	2010
49.4	41.1	9.5	2011
48.1	44.1	7.8	2012
47.3	45.5	7.2	2013
46.4	46.7	6.9	2014
43.7	49.0	7.3	2015
46.2	46.2	7.6	2016
45.5	45.7	8.8	2017

The table shows the shares of water used for household, agricultural, and industrial purposes in Serbia between 2008 and 2017. Data is obtained by dividing the amount of water used in each category by the sum of all water used in the three categories and is measured by million cubic meters and refer to annual freshwater abstraction for household, agricultural, and industrial water use. The vertical axis refers to the interaction coefficients.

**Table 5:** Descriptive statistics for shares of water use in Serbia (2008-17).

	Household Water Use	Agricultural Water Use	Industrial Water Use	*Year of reference*
Mean	23.0	22.4	54.6	
Std Dev	9.8	5.3	14.3	
Min	17.3	13.9	26.7	
Max	41.9	32.1	66.8	

The table shows the descriptive statistics with respect to the shares of water use for household, agricultural, and industrial purposes in Serbia between 2008 and 2017. Std Dev is the standard deviation.

**Table 6:** Descriptive statistics for shares of water use in Bulgaria (2008-17).

	Household Water Use	Agricultural Water Use	Industrial Water Use
Mean	46.7	46.0	7.3
Std Dev	1.9	2.0	1.0
Min	43.6	42.6	6.3
Max	50.2	49.9	9.7

The table shows the descriptive statistics with respect to the shares of water use for household, agricultural, and industrial purposes in Bulgaria between 2008 and 2017. Std Dev is the standard deviation.

**Table 7:** Descriptive statistics for shares of water use in Romania (2008-17).

	Household Water Use	Agricultural Water Use	Industrial Water Use	*Year of reference*
Mean	47.0	44.9	8.2	
Std Dev	2.2	2.6	0.9	
Min	43.7	40.4	7.0	
Max	50.5	49.0	9.5	

The table shows the descriptive statistics with respect to the shares of water use for household, agricultural, and industrial purposes in Romania between 2008 and 2017. Std Dev is the standard deviation.

Going back to the figures, it is possible to identify some of the interactions between water uses that researchers have theoretically conceived as being more persistent. In line with the theory advanced by [7], [8] and [9], and as empirically proved by [10] for China, one could say that there are instances of competition between water uses in some cases. For instance, agricultural and industrial water uses are mostly in a relationship of competition in Bulgaria between 2013 and 2014. This implies that, over this brief period, an increase in the share of agricultural water use generated a negative spillover against the share of industrial water use, and vice versa.

Nevertheless, this should not imply that the emergence of other types of relationship are not conceivable. For example, in Bulgaria and Serbia, agricultural water use functions as a predator between 2008 and 2011, as well as between 2013 and 2017. This could potentially mean that an increase in agriculture water use has a negative impact on household water use, while an increase in household water use could generate an increase in agricultural water use. The fact that an increase in household water use may be able to increase the use of water for agricultural purposes, by creating a positive spillover, is in line with the argument of [28]. According to them, rising incomes and standards of living are likely to precipitate changes in dietary preferences, and therefore to encourage the consumption of goods, such as meat, fish, and dairy products, all of which require a larger amount of water to produce. Their hypothesis builds on the fact that consumption patterns are central drivers in both the increase and reduction of water demands [29].

In addition to these general remarks, the figures reveal some more specific findings. In Bulgaria, for example, except for a brief period of predator-prey relationship between 2010 and 2012 industrial and household water uses mostly alternate relationships of mutualism with competition. In the case of competition, this could mean that an increase in the share of water used for domestic purposes generated a negative spillover on the share for industrial purposes, and vice versa. The same competitive relationships can also be observed for Serbia between 2008 and 2011. Although the causes of these variations can be numerous and cannot directly be inferred from our model, it is sensible to think of some potential factors, especially external ones, which could potentially explain these types of relationships. With respect to industrial and household uses, it is thought that an initial stage of urbanisation would cause them to increase [21]. This mechanism would account for mutualism: more water for industrial use could generate an increase in household use, and vice versa. However, the slowing down of industrial growth and the improvements in water efficiency, brought on by new technological developments, are likely to reduce the amount of industrial water use, to the advantage of household water use [5, 30–32]. This is indeed what happened recently in the US where an intense use of wastewater recycling minimised the demand of water for industrial purposes to the benefit of other uses [33]. An analogous trend also occurred in developing and emerging economies where, at some stage, industrial water use started decreasing due to increases in water prices and stricter environmental regulations (see for e.g. India [34]).

Similar events could therefore explain the alternating relationships of mutualism and competition in the Balkan states of Serbia and Bulgaria, but not of Romania. In fact, in Romania, a predator-prey relationship characterises the interaction between industrial and household water use. While between 2008-2012 and after 2013 household water use is the so-called predator, between these two periods household becomes the prey, in favour of industrial use. Given the high variability over these periods, in both the type and intensity of relationships, explaining the Romanian situation may be complicated.

However, its characteristics may be due to the several changes undergone by the Romanian water management system, following the accession of the country to the European Union in 2007. As reported by the Danube Water Program, in Romania, fifty large water operators replaced smaller water utilities after this accession, so that it would be possible to enhance water-saving and reap efficiency gains by overcoming excessive sector fragmentation [35]. However, one may also note that these efforts have not always coincided with positive results. Despite allocating the highest share of GDP for public investment among the new EU member states in the years 2001-2011 [36], Romania has not significantly improved its infrastructure [37]. Furthermore, the drought in 2007 and the flooding in 2009 and 2010, further complicated the allocation of water and, accordingly, their different interactions.

The same principle could apply to agricultural water use. Agricultural technologies aimed at improving crop water productivity could have the potential to encourage the transfer of larger amounts of water for uses other than agricultural ones [13, 38]. This particular type of reallocation (i.e. from agricultural to household and industrial) is also favoured by researchers as a matter of equity in less industrialised countries [39]. However, such reallocation does not seem to occur in the Balkans in the period considered, due to the fact that interactions between agriculture, and industrial or household uses, appear to be dominated by predator-prey relationships. It is thus very probable that technological developments in agriculture, if any, did not lead to significant water savings in the countries considered. In particular, when looking at Figs 2 and 5 this becomes evident in Bulgaria and Serbia. In both countries, agricultural water use functions as a predator on household use between 2008 and 2011, as well as between 2013 and 2017. Similarly, an increase in the share of water used in the industrial sector implied a decrease in the share of water used for agricultural purposes, while an increase in the share of agricultural water use meant an increase in the share of industrial water use before 2011 and after 2013 in Serbia, and before 2012 in Romania.

With respect to the accuracy of the fitting of the model, following [22], we consider our model to be highly accurate when *MAPE* < 10%; good if 10% < *MAPE* < 20%; reasonable when 10% < *MAPE* < 50%; and inaccurate if *MAPE* > 50%. For Bulgaria, the model appears to be highly accurate for all categories of water uses; namely, MAPE is equal to 2.64846, 2.42373, and 4.14228 for, respectively, agricultural, household, and industrial water uses. This is also true for Romania, for which MAPE is equal to 1.97114, 1.76157, and 2.77147 for the same categories. For Serbia, the model is highly accurate as regards household water use (7.91918) and good for agricultural (10.7317) and industrial (10.3241) water use.

## Conclusion

The interactions among various types of water use may be of different kind and change over periods of time. By exploiting a deterministic model based on differential equations, we identified the specific types of relationships which can occur between different types of water use, as well as the intensity of their interactions. This model has allowed scholars to better understand the varying relationships that exist between different categories [15, 19, 40]. By investigating the interactions in three Eastern European countries, Bulgaria, Romania and Serbia, our article yields two main quantitative findings.

First, it shows that the scarcity of water resources does not automatically translate into competitive relationships, as was already theoretically contended [8, 11]. Secondly, and relatedly, it indicates that interactions are dependent upon contextual factors, which should be taken into consideration by policymakers when implementing water management policies. Specifically, it shows that agricultural, industrial, and household water uses can co-exist in varying relationships of mutualism, competition and predator-prey. This is important for policymakers to understand when they design *ad hoc* institutional arrangements for water allocation in their countries [41]. Knowing, for instance, that an increase in industrial water use is a probable outcome, and that it is likely to have negative effects on the agricultural water use, should invite policymakers to focus on alternatives for improving water efficiency, as well as employing alternative water resources such as wastewater in agriculture [2, 42]. Likewise, having a clearer understanding of water use interactions can be of great relevance for designing effective water transfer policies. Since increasing urban and industrial water uses may motivate water transfers more and more [43], knowing the type of interactions which can exist may facilitate the drafting of a regulatory framework, allowing for inter-sectoral reallocation, that would address the emerging key issues inherent to those different types of relationships (e.g. the legal consequences for the existing right holders and the possible existence of economic externalities).

Therefore, this work should be interpreted as a useful complement, rather than a replacement, to the existing research. Indeed, knowledge of spillovers among water uses seems a necessary component for water policy to improve the management of various water demands; even more so in times of increasing water shortages, when competition among sectors exacerbates the problem, and available water for animals to drink, human beings included, is slowly depleted [2].

On the other hand, we acknowledge that this article has a consciously narrow area of focus; namely, an analysis for Bulgaria, Romania, and Serbia, exploiting their data on water abstraction. Nonetheless, despite its limited geographical scope, this article should encourage further research of the topic, in other European and non-European states, so as to further prove how relationships between water uses are neither of a single type nor constant over time. Furthermore, while doing so, it would be possible to incorporate additional or alternative water sectors, especially the inclusion of the ecological side. Finally, it is possible to imagine that this model could open new avenues of research in water governance such as the analysis of the existing interactions between the 3Es of water management (i.e. efficiency, equity and ecological conservation).
